# Oxford University

**Published:** 1909-09-04

**Authors:** 


### OXFORD UNIVERSITY.

At Oxford University two degrees in medicine can be obtained, the M.B. and the M.D.; similarly in surgery there are two degrees, the B.Ch. and the M.Ch. No candidate is eligible for any of these degrees unless he is a graduate in Arts, that is to say, either an M.A. or a B.A.; in which branch of the Arts that degree has been obtained is a matter of little moment. The usual course of the medical student, who has passed Responsions, is to come up to Oxford and work at the Preliminary Science examinations, which are likely to take him two years or thereabouts, and then to spend a further two years in study for the Final Honour School of Physiology, in which he takes his degree of B.A. He next has to pass examinations in Human Anatomy, and Materia Medica with Pharmacy, after which he proceeds to some town in which the opportunities for clinical study are greater than they are at Oxford, and prepares himself there for his second Professional examination, which comprises the subjects of Medicine, Surgery, Midwifery, Pathology, Forensic Medicine, and Hygiene. When this has been passed, the candidate may supplicate for and obtain the degree of M.B., B.Ch.

Such, in a rough outline, is the programme followed by the majority of medical students at Oxford; but there are not a few exceptions, and these will be indicated in the more detailed account which follows. To begin with, it may be said that the Preliminary Science examinations are in four subjects : Mechanics and Physics, Chemistry, Animal Morphology, and Botany. Examinations in each of these subjects are held at Oxford twice a year, and a fairly high standard of proficiency in them is necessary for a pass. When these Preliminary Science examinations have been safely negotiated, the medical student turns his attention to his Professional examinations. The first of these, commonly known as the "First M.B.," includes Organic Chemistry, Human Physiology, Human Anatomy, and Materia Medica with Pharmacy. Like the Preliminary Science examinations, these subjects may be attacked one by one; and candidates who have obtained a first or second class in Chemistry in the final School of Natural Science are excused Organic Chemistry; while those who have obtained a first or second class in Animal Physiology in their science finals are similarly excused Physiology. It is for this reason that most medical students take up Animal Physiology for their final school, *i.e.* as the subject in which they take their B.A. degree; for by so doing they save more than a year's work. The Second Professional examination is attacked after the candidate has pursued his clinical studies at some large hospital, almost always one of the London hospitals, for two or three years; it is by far the most exacting of the examinations Oxford medical students have to face, for the three subjects of Medicine, Surgery, and Midwifery must be passed together. Pathology or Forensic Medicine, and Public Health may be, and commonly are, taken separately. Before entering for the Second Professional examination (or "Second M.B.") the candidates have to present certificates of attendance at a laboratory course of Practical Pathology and Bacteriology, of having acted as a post-mortem clerk for three months, as surgical dresser for six months, as clinical clerk for six months; they have also to produce certificates to show that they have received instruction in Infectious Fevers, Mental Diseases, and Vaccination, and have attended Labours. The examination is held partly in writing, partly *viva voce*, and (except in Forensic Medicine and Public Health) partly is practical, and is held twice a year.

The degree of M.D. is granted to Bachelors of Medicine of the University who have entered their thirty-ninth term —it should be remembered that there are four terms in the official year—who have presented a dissertation on some medical subject that is approved by the Professors of the Faculty and Examiners for the degree of Bachelor of Medicine for the time being whose subjects are dealt with in it. The degree of M.Ch. is granted to Bachelors of Surgery of the University who have entered their twenty-seventh term and satisfied some other requirements; the examination for this degree is held annually in June.

The University also grants a diploma in Public Health. This is open to all registered medical practitioners, and is in subjects bearing on Preventive Medicine and Public Health—namely, hygiene, general pathology, with special relation to infectious diseases, sanitary law, sanitary engineering, and vital statistics. The examination is held yearly in the Michaelmas term : it is in two parts, which may be taken either separately or together.

The most valuable and valued of the prizes open to

Oxford medical graduates are the Radcliffe Travelling Fellowships. These are three in number, each of the annual value of £200, and tenable for three years, and are awarded, one a year, after an examination held in February. Candidates must have passed all the examinations required for the degrees of B.A. and M.B., and must not have exceeded four years from the time of passing the last examination required for the latter degree. And each candidate must declare that he intends to devote himself during the period of his tenure of the Fellowship to the study of Medical Science, and to travel abroad with a view to that study; at least eighteen months of the three years must be spent abroad. The Radcliffe Prize, founded two years ago by University College, worth £50, is awarded biennially for research in some branch of medical science. The Rolleston Memorial Prize is awarded, biennially, for original research in a number of subjects including Physiology and Pathology; it is open to Cambridge men as well as to Oxford, and no candidate may have exceeded ten years from his matriculation.

